# The Effect of Vitamin D Supplementation on Glycaemic Control in Women with Gestational Diabetes Mellitus: A Systematic Review and Meta-Analysis of Randomised Controlled Trials

**DOI:** 10.3390/ijerph16101716

**Published:** 2019-05-16

**Authors:** Omorogieva Ojo, Sharon M. Weldon, Trevor Thompson, Elisabeth J. Vargo

**Affiliations:** 1 Department of Adult Nursing and Paramedic Science, University of Greenwich, London SE9 2UG, UK; S.M.Weldon@greenwich.ac.uk (S.M.W.); E.Vargo@greenwich.ac.uk (E.J.V.); 2 Barts Health NHS Trust, The Royal London Hospital, Whitechapel Rd, Whitechapel E1 1BB, UK; 3 Department of Psychology, University of Greenwich, London SE10 9LS, UK; T.Thompson@greenwich.ac.uk

**Keywords:** Gestational Diabetes Mellitus, Vitamin D supplementation, meta-analysis, pregnancy, insulin

## Abstract

Vitamin D deficiency is highly prevalent amongst pregnant women and is linked to a range of adverse complications, including gestational diabetes. However, there is no consensus among researchers regarding the impact of vitamin D supplementation in alleviating adverse effects in gestational diabetes. The objective of this systematic review and meta-analysis was to determine whether supplementation of vitamin D given to women with gestational diabetes can promote glycaemic control. EMBASE and PubMed were searched up to November, 2018. The selection criteria included randomised controlled trials of the effect of vitamin D supplementation (1000–4762 IU/day) on pregnant women with gestational diabetes mellitus. Study data and outcome measures (fasting blood glucose, glycated haemoglobin and serum insulin) were extracted from included studies. Random-effects models were used for meta-analyses. Heterogeneity tests, and analysis of the risk of bias were conducted. Most of the studies were graded as having either low risk or moderate risk of bias although two studies had a high risk of bias in the areas of blinding of participants and personnel, and incomplete outcome data. On the other hand, the heterogeneity statistic (I^2^) ranged from 0–41% in the studies included. Five randomised controlled trials were selected for this review and meta-analysis (involving a total of 173 participants supplemented with vitamin D and 153 participants as control drawn from the studies). Vitamin D supplementation was associated with a decrease in fasting blood glucose by a mean of 0.46 mmol/L (−0.68, −0.25) (*p* < 0.001), glycated haemoglobin by a mean of 0.37% (−0.65, −0.08) (*p* < 0.01) and serum insulin concentration by mean of 4.10 µIU/mL (−5.50, −2.71) (*p* < 0.001) compared to controls. This review shows evidence that vitamin D supplementation has the potential to promote glycaemic control in women with Gestational Diabetes Mellitus (GDM). However, due to the limited number of studies in the meta-analysis, the conclusion should be interpreted with caution. Further studies are needed to fully understand the exact mechanism by which vitamin D influences glucose metabolism.

## 1. Introduction

Gestational Diabetes Mellitus (GDM) results from beta cell dysfunction and/or insulin resistance which leads to hyperglycaemia and is unique to pregnancy [1,2,3]. Globally, it affects an estimated 1 in 7 live births [4]. Individuals with GDM are also at risk of developing type 2 diabetes in future and their offspring are at risk of developing childhood obesity and type 2 diabetes later in life [5,6]. A large proportion of cases are seen in low and middle-income countries, where access to maternity care is sparse, exasperating the issue further [4]. Diabetes Mellitus (DM) which antedates pregnancy is not classified as GDM. Instead, these women are diagnosed as having DM and pregnancy [3]. The World Health Organisation (WHO) has recently developed guidelines [5] creating a clear distinction that hyperglycaemia first recognised during pregnancy should be classified either as DM in pregnancy or GDM. There is evidence from a systematic review of cohort studies that hyperglycaemia detected during pregnancy is a risk factor for adverse pregnancy outcomes, including macrosomia of new-born and pre-eclampsia [5].

While well-established risk factors for GDM include high maternal age, maternal overweight or obesity, family history of type 2 diabetes, evidence linking GDM to vitamin D deficiency is growing although it remains inconsistent [6]. Vitamin D deficiency in pregnancy may result in the last trimester of pregnancy due to rapid foetal development, including bone mineralization and calcification [7]. In addition, low exposure to ultraviolet B sunlight, inadequate dietary intake, dark skin colour, veils and obesity, which may result from decreased bioavailability due to increased deposition in fat cells may predispose pregnant women to low vitamin D status [8]. Vitamin D deficiency is common in pregnancy and prevalence varies from 1–90% globally, and with the highest deficiencies seen in the Middle East [7,9,10].

The role of vitamin D in GDM is particularly important as there is emerging evidence that suggests that supplementation of vitamin D can improve insulin sensitivity and glucose intolerance and might control metabolic changes, including hyperglycaemia in pregnancy [9]^.^ According to De-Regil et al. [11], vitamin D helps in maintaining glucose homeostasis by binding and activating vitamin D receptors in the pancreatic beta cells and regulating insulin production in relation to the level of blood glucose. In addition, vitamin D indirectly affects glucose metabolism through its effect on calcium homeostasis [11].

Vitamin D is also known as calciferol which has 2 forms, vitamin D2 (ergocalciferol) and vitamin D3 (cholecalciferol) [9]. Both ergocalciferol and cholecalciferol are pro-hormones which are hydrolysed first to 25-hydroxyvitamin D in the liver which is the main circulating metabolite [12] and then hydrolysed to calcitriol (1, 25-dihydroxyvitamin D-1,25(OH)_2_D) in the kidney [9]. There is evidence that the concentration of the active form, 1,25(OH)_2_D, increases by 100% or more, during pregnancy and this has been partly attributed to an estrogen-dependent increase in vitamin D binding globulin, essential for immune function, thus suggesting extra-skeletal effects of vitamin D [8].

Deficiency of vitamin D is highly prevalent among pregnant women and it has been linked to a range of adverse complications, including GDM [7]. However, despite several studies, there appears to be no consensus among researchers with respect to the role of vitamin D supplementation in terms of need, safety and effectiveness in GDM [13]. In particular, previous systematic reviews and meta-analyses of vitamin D supplementation and gestational diabetes were based on either observational, cohort, or case-control studies and not specifically Randomised Controlled Trials (RCTs) [6,8,14,15,16], or focused on economic implications [17], involved women without gestations diabetes [11,18], were limited in scope [19,20,21], included co-supplementation or had conflicting results and conclusions [22,23]. For example, the Roth et al. [23] review included trials of prenatal vitamin D supplementation given alone or in combination with a co-intervention, while the Akbari et al. [22] review involved studies on post-partum (after delivery) women and co-supplementation with calcium. Agarwal et al. [19] presented a critical review and not a systematic review or meta-analysis, while Wei’s [20] work was an overview of the current literature, and Dornhorst et al. [21] presented a reflection undertaken back in 2002.

In contrast, the present study is a systematic review and meta-analysis of RCT that seeks to determine whether supplementation of vitamin D given alone to women with GDM promotes glycaemic control during the course of pregnancy.

Our research goal is therefore to determine whether vitamin D supplementation is effective in improving glycaemia in women with GDM compared to women with GDM who had no vitamin D supplementation.

## 2. Materials and Methods

This systematic review and meta-analysis were conducted in accordance with the preferred reporting items for systematic reviews and meta-analyses (PRISMA) [24], and followed an a priori, but unpublished protocol available upon request.

### 2.1. Data Sources and Search Strategy

Embase (https://www.embase.com/) and PubMed (https://www.ncbi.nlm.nih.gov/pubmed/) were systematically searched for records from database inception to 20th November 2018 using keywords (Gestational Diabetes Mellitus; Vitamin D supplementation; Randomised Controlled Trial) and subject/medical subject heading (MeSH) (Appendix A) based on Participants, Intervention, Comparator, Outcomes, Study design (PICOS) framework [25]. A hand reference search of the available literature was also performed. All citations from database searches were exported to Excel and duplicates were removed.

### 2.2. Eligibility Criteria

Inclusion criteria were: (1) The use of only vitamin D supplementation; (2) a population of women with GDM irrespective of age or the number of previous births; (3) studies carried out only during pre-natal period; (4) an RCT design with an intervention and no-intervention (i.e., control group or placebo); (5) a definition of GDM where a diagnosis was based on international and local criteria (6) papers written in English.

Exclusion criteria were: Any study that was not an RCT; studies involving healthy pregnant women without GDM; studies including women with pre-existing type 1 or type 2 diabetes diagnosed before pregnancy; studies involving vitamin D and co-supplementation, and studies carried out after delivery or post-partum.

### 2.3. Study Selection

One author (E.J.V.) scanned all article titles and abstracts for eligibility, and then performed a second search of the full text for potentially eligible articles retained from the first search to reach a final list of included studies. This process was replicated by another author (O.O.). All remaining authors spot-checked the included and excluded abstracts for consistency with the eligibility criteria.

**Figure 1:**
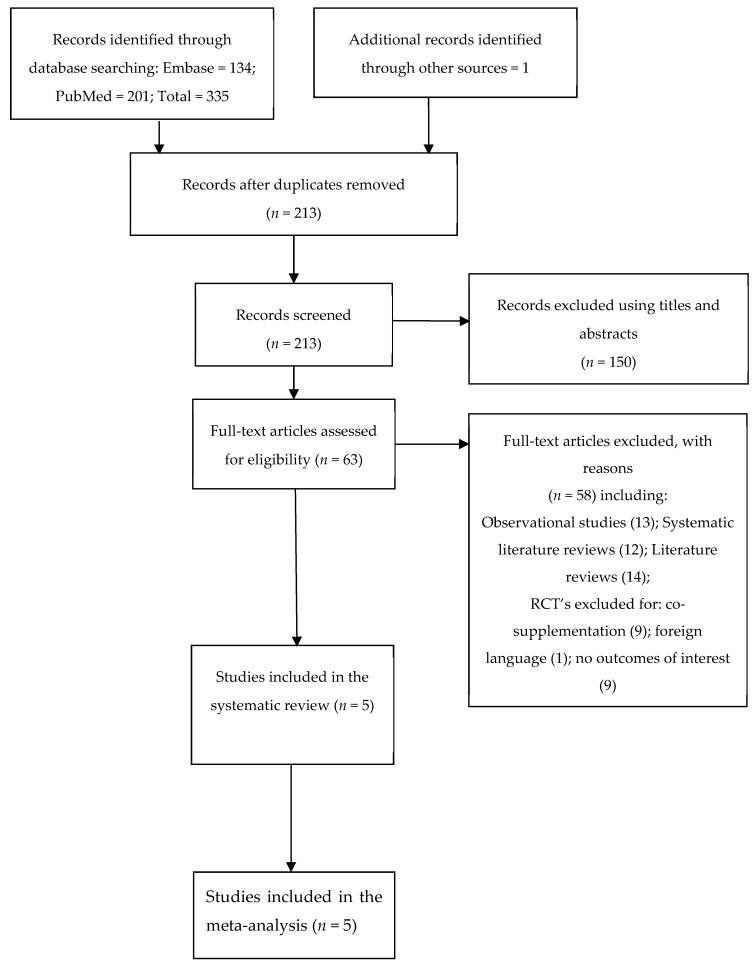
Preferred reporting items for systematic reviews and meta-analyses (PRISMA) study selection.

The search strategy conducted produced 335 records. One additional record was identified through a hand reference search. 213 records remained once duplicates were removed. After an inspection of title and abstracts, a further 150 records were excluded. Of the 63 extracted records, five papers were included in the systematic review and meta-analysis (Figure 1).

### 2.4. Quality Evaluation

The Critical Appraisal Skills Programme (CASP) checklist for RCTs was used to evaluate the quality of the studies included in the review [26]. The Cochrane Collaboration tool was used to assess the risk of bias of each included study. Two authors (S.M.W. and E.J.V.) assessed each study independently against the seven criteria, including selection bias, performance bias, personal bias, detection bias, attrition bias and reporting bias [27]. Assessments were made using the information provided in the published paper only.

### 2.5. Information Extraction

Information was extracted from the studies into an extraction table (Table 1); that documented study year, country, type of study, study dates, length of study, sample size, mean age of mother, intervention, outcome measures of interest, risk of biases, study results and the criteria for defining GDM. Table 2 provided the estimated average daily intake of Vitamin D and pre-intervention Vitamin D levels in blood/serum. The data were extracted and crosschecked by three authors.

For the meta-analysis, the following outcome measures (metabolic parameters) of interests were extracted (where available) from each study:

- fasting plasma glucose (FPG) (mmol/L)
- glycated haemoglobin (HbA1c) (%)
- serum insulin concentration (µIU/mL)

Fasting plasma glucose and glycated haemoglobin were selected as outcomes of interest as these are short- and longer-term measures of glycaemic control respectively. These parameters have also been linked to neonatal and maternal outcomes, including pre-eclampsia and birthweight [28]. In addition, the role of serum insulin in glucose homeostasis has been the subject of intense research.

### 2.6. Data Synthesis and Statistical Analyses

RevMan (Review Manager, 5.3, (Copenhagen, Denmark) [29] was used for meta-analysis and sensitivity analysis. Since the time, location, population of studies and dosage of vitamin D supplementation were varied, this is likely to result in heterogeneity in effect size and thus a random effects models were used to pool the data, rather than a fixed-effect model which assumes a single, common effect size across studies. Forest plots were depicted to visually assess the effect sizes, the difference in means and 95% confidence interval (CIs) across the studies.

Heterogeneity across studies was assessed with the Cochrane Q test and a significance level of *p* < 0.10 was used to determine statistical significance [27]. In addition, the *I*^2^ statistic (from 0 to 100%) with higher values representing greater inconsistency in effect size across studies was computed [30]. The test for publication bias would have been conducted using a funnel plot and Eggers test if we had ten or more studies with an outcome of interest.

## 3. Results

### 3.1. Study Selection

All studies included were published during 2012–2016. Of the five studies included in the systematic review, three of the studies were conducted in Iran and two in China. Study length varied between 4–16 weeks (Table 1). The sample size for the vitamin D supplemented group in the studies selected ranged from 24–48 while for the control group it ranged from 20–49. The mean age of the mother taking part in the study was 28 years of age. GDM screening was conducted anytime between 13 weeks and 28 weeks gestation. Vitamin D supplementation intervention amounts, and times given varied across all studies (Table 2), however a pattern of 50,000 IU given at least twice within the study emerged. Administration of vitamin D supplementation was orally (tablets and vitamin D supplemented yoghurt) and by injection. Outcome measures of interest differed across studies; however, FPG, glycated haemoglobin and serum insulin concentration were the most regularly reported. The majority of studies reported a beneficial effect of vitamin D on GDM.

### 3.2. Data Inclusion Decisions and Discrepancies

Zhang et al. [33] dosed participants according to low, medium and high levels of Vitamin D. This posed an issue regarding what dosage to use for our meta-analysis. We emailed the authors of the article, but, received no response. We, therefore, chose the medium dose results to include in this study, due to it having the closest similarities to the other studies of interest. Another issue in this study was a discrepancy between the results presented in the text and those presented in the graphs. There appeared to have been a mix up between the control and low dosage group for fasting plasma glucose, and the medium and high dosage groups for serum insulin concentrations. The differences were minimal and we, therefore, decided to use the text results over the graph results. Standard deviations were not presented in the text and therefore the graphs were converted in Web Plot Digitiser in order to obtain these.

### 3.3. Assessment of The Risk of Bias

**Figure 2:**
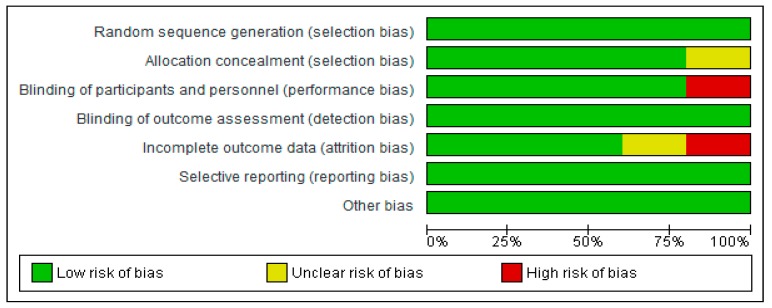
Risk of bias graph per type of bias assessed.

**Figure 3:**
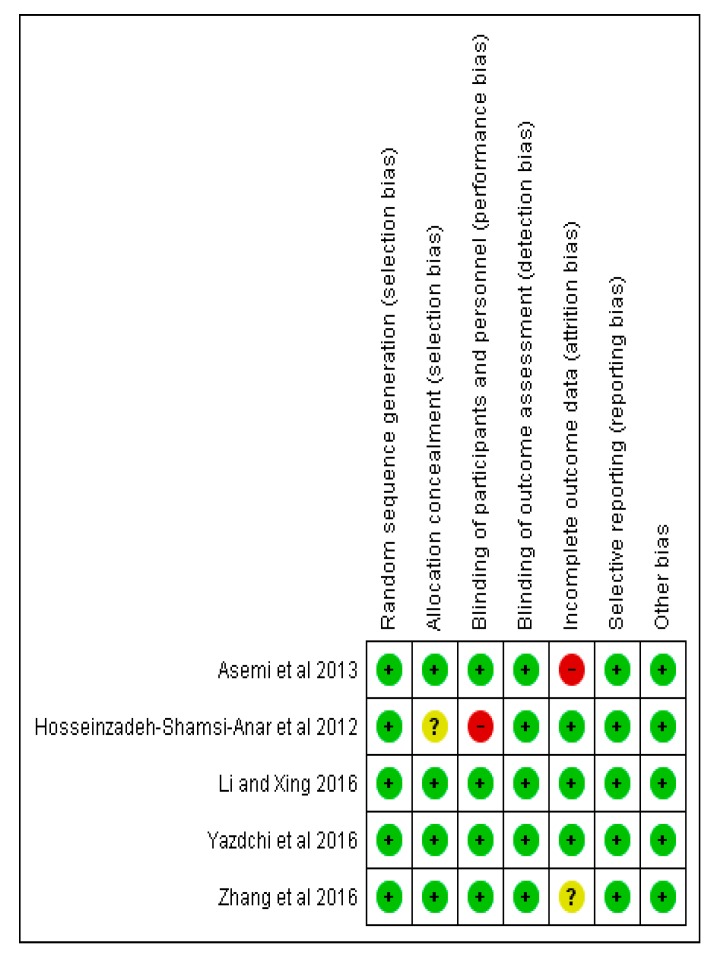
Risk of bias summary for the studies assessed.

Results of the Cochrane risk of bias assessments (Figure 2 and Figure 3) present an overall low risk of bias, especially in relation to random sequence generation. A slightly higher risk was reported for performance bias, and attrition bias. Unclear risks were related to selection bias and attrition bias. Publication bias could not be conducted, due to limited data.

### 3.4. Effect of Vitamin D Supplementation During Pregnancy on Metabolic Parameters

**Figure 4:**
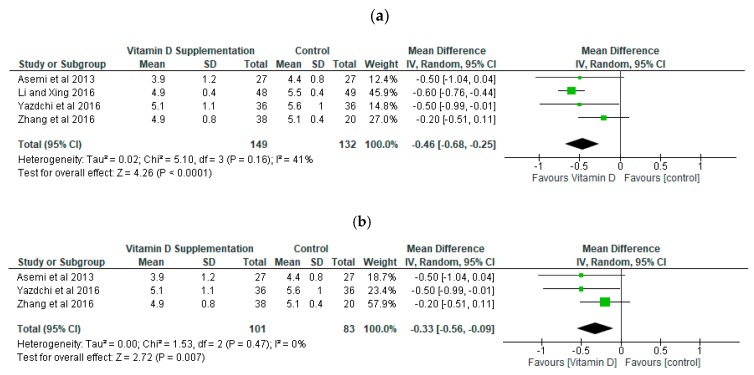
Forest Plot of Fasting Blood Glucose (mmol/L). (**a**) Meta-analysis; (**b**) Sensitivity analysis.

**Figure 5:**
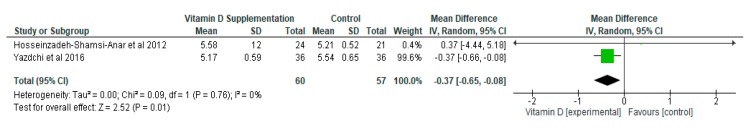
Forest plot of glycated haemoglobin (%).

**Figure 6:**
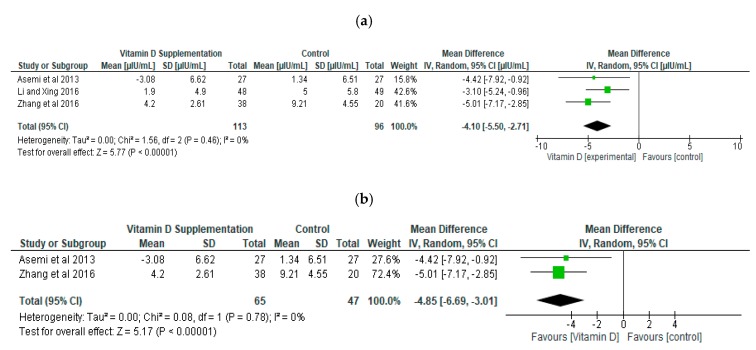
Forest plot of serum insulin concentration µIU/mL. (**a**) Meta-analysis; (**b**) sensitivity analysis.

We performed meta-analysis and sensitivity analysis by removing studies in turn in respect of the fasting blood glucose (Figure 4a,b) and serum insulin concentration (Figure 6a,b). Overall the mean difference between the intervention and control group was statistically significant in respect of fasting blood glucose (*p* < 0.001) and glycated haemoglobin (*p* < 0.01) (Figure 5). This result indicates that vitamin D supplementation during pregnancy decreases fasting blood glucose by a mean of 0.46 mmol/L (−0.68, −0.25). The level of heterogeneity analysed across the studies was not significant (*p* = 0.16) with a low *I*^2^ (41%). The sensitivity analysis also showed a significant improvement (*p* = 0.007) in the group supplemented with vitamin D compared to the control group in relation to fasting blood glucose (Figure 4b). A further sensitivity analysis involving the exclusion of the other studies in turn demonstrated similar findings to the results of the meta-analysis (*p* < 0.01)

For glycated haemoglobin, the two studies included had contrasting findings. However, the mean difference between the vitamin D supplementation group and control was also significant (*p* < 0.01) with the vitamin D supplementation decreasing glycated haemoglobin by a mean of 0.37% (−0.65, −0.08) (Figure 5).

Regarding the effect of vitamin D supplementation during pregnancy on serum insulin concentration, the meta-analysis results of three studies revealed a decline by mean of 4.10 µIU/mL (−5.50, −2.71), which is statistically significant at *p* < 0.001 (Figure 6a). Heterogeneity was insignificant (*p* = 0.46) with an *I*^2^ of 0%. The sensitivity test of serum insulin concentration revealed a significant decline (*p* < 0.001) in the group supplemented with vitamin D compared with control group with a mean difference of −4.85 µIU/mL (Figure 6b). Similar findings (*p* < 0.001) were observed by removing other studies in turn.

## 4. Discussion

The results of the systematic review and meta-analysis show that vitamin D supplementation has a significant effect on serum insulin and blood glucose parameters. These results were confirmed by the sensitivity analyses. In particular, there were significant decreases in fasting blood glucose, glycated haemoglobin and serum insulin concentrations in women with GDM compared with the control groups. These findings provide more robust evidence compared with the results of previous systematic reviews and meta-analysis of observational studies involving vitamin D and GDM [6,14,16]. These earlier studies indicated a consistent association between vitamin D deficiency and increased risk of GDM and that supplementation of vitamin D could ameliorate the condition, but, it remains unclear whether this association is actually caused by vitamin D. The present review addresses the limitations of previous observational studies by including only RCTs which were needed to fully elicit the impact of vitamin D supplementation on women with GDM. Treatments in observational studies are not allocated by chance and thus are likely to give rise to the imbalance between the groups being compared [35].

The present review is also different from previous reviews which were based on RCTs. Roth et al.’s [23] review included vitamin D supplementation given in combination with a co-intervention, while Akbari et al. [22] in their review selected studies that combined vitamin D with calcium (Asemi et al. [36]) and studies (Mozffari-Khosravi et al. [37]; Valizadeh et al. [38]) that involved post-partum women. In contrast, the present review is unique and the first that is based on RCTs of vitamin D supplementation given alone and focuses on women with gestational diabetes and their glycaemic outcomes before parturition. Therefore, while Akbari et al. [22] found no significant differences between the groups supplemented with vitamin D and control in terms of fasting blood glucose, glycated haemoglobin and insulin levels, the current review found significant (*p* < 0.05) improvement in the vitamin D supplemented group compared with control in relation to these parameters. According to Hodson et al. [2], the sensitivity to insulin decreases progressively during pregnancy and returns swiftly to normal following delivery. Thus, the inclusion of studies involving participants who had delivered may have influenced the results of the Akbari et al. [22] study when compared to our review which included only pre-natal women with GDM.

While previous studies have tended to focus primarily on the broader association between vitamin D and gestational diabetes, maternal and/or neonatal endpoints and less on metabolic parameters, the present review sees these outcomes as interrelated. For example, the metabolic parameters, including fasting blood glucose and glycated haemoglobin have been reported in some studies as the major factors in GDM that influence clinical outcomes, such as birthweight. Therefore, their evaluation is critical in understanding the pathways of the clinical manifestations of maternal and neonatal outcomes. There is evidence that glycated haemoglobin can be used to estimate the risk of pregnancy complications [39]. Furthermore, the Hyperglycaemia and Adverse Pregnancy Outcome (HAPO) study involving 25,505 pregnant women has demonstrated strong associations of maternal glucose levels below the diagnosis level for diabetes with increased birthweight [28].

Rudnicki and Molsted–Perdersen [40] evaluated the effect of 1,25-dihydrovitamin D supplement on glucose metabolism in 12 women with gestational diabetes. The results showed that intravenous 1,25 dihydroxyvitamin D3 significantly decreased fasting blood glucose levels in GDM although the differences were not significant following 14 days of oral vitamin D supplementation. The authors concluded that the administration of vitamin D3 tends to decrease fasting blood glucose. Similarly, Asemi et al. [41] found that vitamin D supplementation over nine weeks significantly decreased fasting plasma glucose, insulin concentrations and significantly increased insulin sensitivity. The findings of the current systematic review and meta-analysis support these earlier results.

Rudnicki and Molsted – Perdersen [40] noted that the level of insulin decreased after intravenous and oral treatment with vitamin D. Therefore, the decrease in the levels of fasting blood glucose and glycated haemoglobin observed in the current review may be due to the action of vitamin D through the increased cellular absorption of glucose either directly or by enhancing the action of insulin [40]. In other words, the lower insulin levels detected in the current review in the vitamin D group compared with the control group would suggest that the mechanism of improved glucose tolerance was not due to increased production of insulin, but potentially from an increased sensitivity to the action of insulin [9]. Vitamin D has been shown to improve insulin sensitivity in target cells (liver, skeletal muscle and adipose tissue) and protect them from the detrimental effects of immune attack [42]. The mechanism of vitamin D action in glucose homeostasis in GDM may involve stimulating the expression of insulin receptors in peripheral tissues, thus, regulating the uptake of glucose [43,44]. Insulin mediated glucose uptake is dependent on calcium; therefore, the vitamin D status may indirectly influence glucose transport in target tissues through the regulation of intracellular calcium [14,44]. The different actions of vitamin D may explain why previous studies have suggested that the effect of vitamin D on glucose may be through its influence on insulin secretion in the pancreatic beta cells [40,45]. Based on the different views on the role of vitamin D in glucose homeostasis, it is safe to suggest that further studies are needed to fully understand the exact mechanism by which vitamin D influences glucose metabolism.

Drawing from the results of this review and meta-analysis, it could be argued that vitamin D has a role in maintaining glucose homeostasis and thus has clinical and public health implications. In this regard, the National Institute for Health and Care Excellence (NICE) [46] has recommended increased access to vitamin D supplements for pregnant women to ensure that they meet the Reference Nutrient Intake. NICE [46] recommended that the Department of Health should work with manufacturers to ensure that vitamin D supplements providing the Reference Nutrient Intake of 10 micrograms per day of vitamin D for pregnant women are available. Boucher et al. [47] also recommended that women should be provided with vitamin D supplements as currently recommended nationally for pregnancy. In addition, the Royal College of Obstetricians and Gynaecologists (RCOG) [48], noted that, daily vitamin D supplementation with oral cholecalciferol or ergocalciferol is safe in pregnancy and that vitamin D 10 microgram (400 units) a day should be provided for all pregnant women in line with national guidelines. Pregnant women should also be encouraged to receive adequate nutrition which is best accomplished through the consumption of a healthy balanced diet [49,50].

### Limitations

A strength of this review is its focus on RCTs. Although most of the studies were graded as having either low risk or moderate risk of bias while two studies had a high risk of bias in the areas of blinding of participants and personnel, and incomplete outcome data, within each RCT included there was variation in the amount, route and timings of vitamin D dosages given. Outcomes were based on studies conducted in only two countries, and RCT studies available for meta-analysis were very few and the sample sizes were small. In addition, the exclusion of studies not written in English may have limited the number of studies included in this review. Some discrepancies were found in Zhang et al. [33] study. These would affect the validity of the results of this review, thus more studies are needed to build on the evidence base.

## 5. Conclusions

This review has demonstrated that vitamin D supplementation has the potential to promote glycaemic control in women with GDM. However, due to the limited number of studies in the meta-analysis, the conclusion should be interpreted with caution. Further studies are needed to fully understand the exact mechanism by which vitamin D influences glucose metabolism.

## Figures and Tables

**Table 1:** Data extraction table for all included studies.

Citation	Country	Type of Study	Study Dates	Length of Study	Sample Size (Intervention, Control-Placebo)	Mean Age of Mother	Time of Vitamin D Intervention	Intervention	Outcome Measures of Interest	Risk of Biases	Study Results
#Asemi et al. (2013a) [31]	Iran	Randomized, double-blinded, placebo controlled clinical trial	Jan 2013–April 2013	6 weeks	54 (27,27)	31.5 ± 6.1 years	At 24–28 weeks of gestation	50,000 IU Vitamin D_3_ supplements at baseline and at day 21	FPG, SI concentration	6/7 low risk 1 high risk for attrition bias	Change from baseline: Vitamin D group versus the control FBG −17.12 ± 14.84 compared with −0.96 ± 16.64 mg/dL; *p* < 0.001- SI Concentration −3.08 ± 6.62 compared with 1.34 ± 6.51 µIU/mL; *p* = 0.01 ANOVA
#Li and Xing, (2016) [32]	China	Randomized Study Design	Between early November and late February each year from 2010 to 2014	16 weeks	97 (48,49)	24–32 years old	At 13 weeks of gestation	2 servings (200g) of supplemented yogurt per day (500 IU vitamin D_3_ per serving)	FPG, SI concentration	7/7 low risk	Change from baseline: Vitamin D group versus the control FBG −9.9 ± 7.2 compared with 2.9 ± 7.6 mg/dL; *p* = 0.04 SI Concentration −1.9 ± 4.9 compared with 5.0 ± 5.8 µIU/mL; *p* = 0.03 *t*-test
##Zhang et al. (2016) [33]	China	Randomized, double-blind placebo-controlled clinical trial	Sept 2009–Nov 2014	4 weeks	133 (38,20) **	29.8 ± 4.7 years	At 24–28 weeks of gestation	Low dosage: 200 IU Vitamin D supplement daily Medium dosage: 2000 IU daily for 25 days High dosage: 4000 daily for 12.5 days	FPG, SI concentration	6/7 low risk 1 unclear risk for attrition bias	FBG Low: 96.12; Medium: 88.59; High: 84.73 vitamin D supplementation compared with control: 92.49 mg/dL; *p* > 0.05 SI Concentration Medium: 5.01; High: 4.2 vitamin D supplementation compared with control: 9.21 IU/mL; *p* < 0.01 Non-parametric tests
###Yazdchi et al., (2016) [34]	Iran	Randomized, double-blinded, placebo-controlled clinical trial	July 2013–Sept 2014	8 weeks	72 (36,36)	31.88 ± 4.0 years	At 24–28 weeks of gestation	4 oral treatments of 50,000 IU of Vitamin D_3_ (one capsule every 2 weeks)	FPG, SI concentration HbA1c	7/7 low risk	Change from baseline: Vitamin D group versus the control FBG −4.72 ± 13.99 compared with 5.27 ± 9.93 mg/dL; *p* = 0.01 SI Concentration 1.80 (−1.67–3.77) compared with −0.45 (−1.07–1.35) µIU/mL; *p* = 0.23 HbA1c −0.18% ± 0.48% compared with 0.17% ± 0.39%; *p* = 0.02 ANCOVA
####Hosseinzadeh-Shamsi-Anar et al. (2012) [7]	Iran	Randomized clinical trial	Study dates not provided	12 weeks	45 (24,21)	30.7 ± 6.2 years	At 24–28 week gestation	Intramuscular 300,000 IU of vitamin D	HbA1c	5/7 low risk 1 unclear risk for selection bias 1 high risk for performance bias	Vitamin D group versus control HbA1c 5.58% ± 12 compared with 5.21±0.52% *p* = 0.2 *t*-test

Abbreviations: ANCOVA (analysis of covariance); ANOVA (analysis of variance); FBG (fasting blood glucose); HbA1c (glycated haemoglobin); SI (serum insulin). Criteria for Defining GDM (# = Criteria set by the American Diabetes Association; ## = Local criteria; ### = International Association of Diabetes and Pregnancy Study groups criteria; #### = Carpenter and Coustan Criteria for screening tests for gestational diabetes). ** Results for the medium dosage used by the authors (see 3.2 Data inclusion decisions and discrepancies).

**Table 2:** Estimated Average Daily Intake of Vitamin D and Pre-Intervention Vitamin D Levels in Blood/Serum.

Citation	Interventions	Estimated Average Intake of Vitamin D Per Day IU/day	Pre-Intervention Vitamin D Level in Blood/Serum
Asemi et al. (2013a) [31]	Vitamin D	4762	20.44 ± 14.31 *
	Control	Placebo	20.41 ± 13.43 *
Li and Xing, (2016) [32]	Vitamin D supplemented Yoghurt	1000	16.8 ± 4.6 *
	Plain Yoghurt	Plain Yoghurt	16.2 ± 3.4 *
Zhang et al. (2016) [33]	Vitamin D	2000	Actual values not stated. Vit. D deficiency (<20 ng/mL)
	Control	Placebo	Actual values not stated. Vit. D deficiency (<20 ng/mL)
Yazdchi et al., (2016) [34]	Vitamin D	3333	9.54 (Median) (6.12–15.94) (25th and 75th percentile)
	Control	Placebo	9.02 (Median) (7.29–14.70) (25th and 75th percentile)
Hosseinzadeh-Shamsi-Anar et al. (2012) [7]	Vitamin D	3333	24.25 nmol/L (Median) (13.3–202.4) (Min–Max)
	Control	No Vitamin D	25.30 nmol/L (Median) (12.8–137.2) (Min–Max)

Note: * ng/mL (Mean ± SD).

## Appendix A. Search Terms

### Embase

Search date: up to 20 November 2018

Database: Embase <1974 to current >

1. exp pregnancy diabetes mellitus/or Gestational diabetes.mp. or GDM.mp. or gestational diabetes mellitus.mp. or diabetes mellitus, gestational.mp. or diabetes in pregnancy.mp (33032).
2. exp vitamin D/or Vitamin D.mp. or 25- hydroxyvitamin D.mp. or 25OHD.mp. or calciferol.mp. or ergocalciferol.mp. or cholecalciferol.mp (144930).
3. exp randomized controlled trial/or Randomised controlled trial.mp. or Controlled clinical trial.mp. or Randomized.mp. or Placebo.mp (1357496).
4. 1 and 2 and 3 (138).
5. limit 4 to human (134).

### Pubmed

Search: up to 20 November 2018

#1 Gestational diabetes OR GDM OR gestational diabetes mellitus OR diabetes mellitus, gestational OR diabetes in pregnancy OR Diabetes, gestational [MeSH] OR pregnancy in diabetics [MeSH] (37699).
#2 Vitamin D OR 25-hydroxyvitamin D OR 25OHD OR calciferol OR ergocalciferol OR cholecalciferol OR Vitamin D [MeSH] (79485).
#3 Randomised controlled trial OR Controlled clinical trial OR Randomized OR Placebo OR Drug therapy OR randomly OR trial OR groups OR “Randomized Controlled Trials as Topic” [MeSH] (5428822).
#4 Animals [MeSH] NOT Humans [MeSH] (4517123).
#5 #1 AND #2 AND #3 (208).
#6 #5 NOT #4 (201).
